# Predicting anxiety from wholebrain activity patterns to emotional faces in young adults: a machine learning approach

**DOI:** 10.1016/j.nicl.2019.101813

**Published:** 2019-04-03

**Authors:** Liana C.L. Portugal, Jessica Schrouff, Ricki Stiffler, Michele Bertocci, Genna Bebko, Henry Chase, Jeanette Lockovitch, Haris Aslam, Simona Graur, Tsafrir Greenberg, Mirtes Pereira, Leticia Oliveira, Mary Phillips, Janaina Mourão-Miranda

**Affiliations:** a Centre for Medical Image Computing, Department of Computer Science, University College London, United Kingdom; b Department of Physiology and Pharmacology, Federal Fluminense University, Niteroi, Brazil; c Department of Psychiatry, Western Psychiatric Institute and Clinic, University of Pittsburgh Medical Center, University of Pittsburgh, Pittsburgh, United States; d Department of Psychological Medicine, Cardiff University, Cardiff, United Kingdom; e Max Planck University College London Centre for Computational Psychiatry and Ageing Research, University College London, United Kingdom

**Keywords:** RDoC, Anxiety, Depression, fMRI, Pattern recognition, Pattern regression analysis, Machine learning, Faces, MVPA

## Abstract

**Background:**

It is becoming increasingly clear that pathophysiological processes underlying psychiatric disorders categories are heterogeneous on many levels, including symptoms, disease course, comorbidity and biological underpinnings. This heterogeneity poses challenges for identifying biological markers associated with dimensions of symptoms and behaviour that could provide targets to guide treatment choice and novel treatment. In response, the research domain criteria (RDoC) (Insel et al., 2010) was developed to advocate a dimensional approach which omits any disease definitions, disorder thresholds, or cut-points for various levels of psychopathology to understanding the pathophysiological processes underlying psychiatry disorders. In the present study we aimed to apply pattern regression analysis to identify brain signatures during dynamic emotional face processing that are predictive of anxiety and depression symptoms in a continuum that ranges from normal to pathological levels, cutting across categorically-defined diagnoses.

**Methods:**

The sample was composed of one-hundred and fifty-four young adults (mean age=21.6 and s.d.=2.0, 103 females) consisting of eighty-two young adults seeking treatment for psychological distress that cut across categorically-defined diagnoses and 72 matched healthy young adults. Participants performed a dynamic face task involving fearful, angry and happy faces (and geometric shapes) while undergoing functional Magnetic Resonance Imaging (fMRI). Pattern regression analyses consisted of Gaussian Process Regression (GPR) implemented in the Pattern Recognition for Neuroimaging toolbox (PRoNTo). Predicted and actual clinical scores were compared using Pearson's correlation coefficient (r) and normalized mean squared error (MSE) to evaluate the models' performance. Permutation test was applied to estimate significance levels.

**Results:**

GPR identified patterns of neural activity to dynamic emotional face processing predictive of self-report anxiety in the whole sample, which covered a continuum that ranged from healthy to different levels of distress, including subthreshold to fully-syndromal psychiatric diagnoses. Results were significant using two different cross validation strategies (two-fold: r=0.28 (p-value=0.001), MSE=4.47 (p-value=0.001) and five fold r=0.28 (p-value=0.002), MSE=4.62 (p-value=0.003). The contributions of individual regions to the predictive model were very small, demonstrating that predictions were based on the overall pattern rather than on a small combination of regions.

**Conclusions:**

These findings represent early evidence that neuroimaging techniques may inform clinical assessment of young adults irrespective of diagnoses by allowing accurate and objective quantitative estimation of psychopathology.

## Highlights

• The RDoC advocate a dimensional approach which omits any disorder thresholds.
• Combine fMRI and pattern regression to predict dimensional psychiatry symptoms.
• Patterns of neural activity predict anxiety which covered a continuum.
• Neuroimaging inform clinical assessment of adults from healthy to different levels of distress.

## 1 Introduction

Almost one-fifth of all 18–25-year olds seek help from mental health professionals for psychological distress, which ranges from anxiety and depressive symptoms to personality traits, functional disabilities and behavioural problems (SAMHSA, 2007). Identifying biomarkers reflecting pathophysiological processes associated with these dimensions of behaviour has the potential to provide biologically-relevant targets that can guide treatment choice and novel treatment development (Phillips and Frank, 2006; Hasler et al., 2006). While studies using neuroimaging techniques have the potential to identify these biomarkers, most neuroimaging studies in youth suffering from anxiety and depression have focused on identifying group differences in neural circuitry associated with the categorical diagnoses (Stein et al., 2007; Blair et al., 2008; Stuhrmann et al., 2011; Demenescu et al., 2011; Duval et al., 2015). This approach is helpful for making regionally specific inferences about abnormalities in brain function and structure that may be associated with anxiety and depression; however, such an approach describes differences at the group level and do not enable predictions *at the individual level*, which is more desirable in clinical practice, where physicians need to make decisions about individuals.

Over the last ten years, machine learning approaches, such as pattern recognition, also known in the neuroimaging field as Multivoxel Pattern Analysis (MVPA), have been increasingly used to identify multivariate patterns in neuroimaging data that enable prediction at the individual subject level (for reviews, see Haynes and Rees, 2006; Cohen et al., 2011, Marquand et al., 2016, Janssen et al., 2018). These techniques are promising for identifying neurobiological measures that can predict scores on a given dimension, for example dimensional measures of anxiety and depression. In the context of pattern recognition, the term “predict” means that once the model has learned a relationship between a set of patterns (e.g. multivariate patterns of brain activation) and labels (e.g. a clinical score), given a new pattern (e.g. brain activation from a new subject) it can predict its label. In other words, in pattern recognition analysis the model performance (e.g. predictive accuracy for classification or mean squared error for regression) is evaluated on new data that was not used to train or fit the model. For example, fMRI and pattern recognition techniques have been used to identify relationships between patterns of brain activity and continuous measures of behaviour or symptoms. Such information was then used to predict individual-level scores on symptom/behavioural measures in a set of new individuals (Stonnington et al., 2010; Cohen et al., 2011; Portugal et al., 2016; Fernandes et al., 2017). As a multivariate approach, pattern recognition also has the potential to be more sensitive to spatially distributed and subtle effects in the brain than a standard mass-univariate analysis, potentially providing a more powerful approach for studies of subclinical populations in which less severe alterations are generally observed. To date, there are few neuroimaging studies that applied pattern recognition approaches to predict current or future clinical scores in young adults seeking help for psychological distress (Chase et al., 2017; Greenberg et al., 2017).

Currently the standard diagnostic approach for people seeking help for psychological distress is based on the Diagnostic and Statistical Manual of Mental Disorders (DSM) and the International Classification of Diseases (ICD), which relies on specific symptom criteria for establishing a categorical diagnosis. Although these systems have contributed greatly to the reliability of psychiatric diagnoses made for research and clinical purposes, their categories and criteria were formulated before modern neuroscience and perhaps do not reflect the organization of brain circuits and their associated behaviours (Morris and Cuthbert, 2012). Yet, it is becoming increasingly clear that pathophysiological processes underlying such disease definitions are heterogeneous (Hyman, 2007; Insel and Cuthbert, 2015). For example, some studies have shown that a specific drug or psychotherapy intervention can be successful in a certain patient subgroup and unsuccessful in another patient subgroup labelled with an identical diagnosis (Gabrieli et al., 2015). This variability in treatment response, which is not understood and not simply a consequence of disease severity, suggests that there are clinically important neurobiological differences among patients sharing a diagnostic label. High comorbidity rates also contribute to this debate, raising questions about the core features of a specific diagnosis and poses problems for diagnosis and treatment (Sanislow et al., 2010). The ongoing discussion surrounding psychiatric nosology reflects well-acknowledged difficulties in finding biological markers that predict current disease state or future outcomes with sufficient sensitivity and specificity to be clinically useful (Kapur et al., 2012; Insel and Cuthbert, 2015).

Motivated by the fact that most findings from neuroscience and genetics either link to many different syndromes or distinct subgroups within syndromes, but not to DSM diagnostic categories of psychiatric disorder, the research domain criteria (RDoC) (Insel et al., 2010) was developed to advocate a dimensional approach to understanding the pathophysiological processes underlying psychological distress and other mental health problems. An important contribution is a shift away from symptoms and towards conceptualizing psychopathology as spanning multiple domains of functioning and across multiple units of analysis. An important assumption of the RDoC is that psychopathologies are heterogeneous phenomena involving multiple pathophysiological processes, which make them difficult targets for reduction (Cuthbert and Insel, 2013). It is also important to highlight that the RDoC framework intentionally omits any disease definitions, disorder thresholds, or cut-points for various levels of psychopathology. Furthermore, these behavioural changes exhibited by individuals with mental health disorders may be the tip of an iceberg—a late manifestation of a change that has been occurring in the brains of people who were still considered psychiatrically “healthy”, thereby suggesting that these disorders may be better conceptualized as “brain disorders”.

In this context, Oathes et al. (2015) used resting-state functional magnetic resonance imaging (fMRI) and principal component data reduction to elucidate the relative contributions of categorial and dimensional formulations implicated in anxiety and depression disorders. They showed that general distress, measured by a self-rated scale, is positively associated with limbic and paralimbic signal amplitudes, whereas the presence of a depressive disorder diagnosis drives signal down across these neural regions. They also showed a positive association between anxious arousal and connectivity between the anterior cingulate cortex and the ventral striatum, whereas the presence of a depressive disorder diagnosis was associated with reduced connectivity among these regions. While the authors suggested that using a single conceptual framework (i.e. categorical diagnoses or symptom dimensions) provides an incomplete mapping of psychopathology to neurobiology, they argued that symptoms are especially strong predictors of resting-state connectivity. Supporting the concept behind the RDoC framework, some studies employed clustering methods to stratify individuals with psychiatric disorders across conventional diagnostic categories (Olino et al., 2010, Lewandowski et al., 2014, Kleinman et al., 2015, for a review see Marquand et al., 2016). For instance, Drysdale et al. 2016 used resting state fMRI in a large multisite sample and showed that patients with depression could be subdivided into four neurophysiological subtypes defined by distinct patterns of dysfunctional connectivity in limbic and frontostriatal networks. However, more recently, Dinga et al., 2018 attempted to replicate the results of Drysdale et al. 2016 without success. In fact, the authors did not find clearly distinct subtypes of depression and argued that the evidence for the existence of the distinct resting state connectivity-based subtypes of depression is weak and should be interpreted with caution.

Nevertheless, to the best of our knowledge, no study has applied pattern regression analysis to find brain signatures in fMRI task data that are predictive of anxiety and depression in a continuum that ranges from normal to pathological levels, cutting across categorically-defined diagnoses. In the present study, we applied pattern regression analysis to determine whether we could find brain signatures during dynamic emotional face processing, a task that has previously been shown to elicit abnormal activity in widespread neural regions in individuals with anxiety and depressive disorders, related to anxiety and depression symptoms (Hafeman et al., 2017; Greenberg et al., 2017; Manelis et al., 2015). We recruited young adults (18–25 years) self-identified as distressed irrespective of the presence or absence of a psychiatric diagnosis and healthy individuals (see Materials and Methods for definition of these groups). This approach thereby allowed us to include young adults across a wide range of anxiety and depressive symptoms. In summary, the aim of the present study was to combine fMRI and pattern regression analysis to determine, as a proof of concept, whether patterns of brain activity during dynamic emotional face processing could accurately predict anxiety and depressive symptoms in a sample of 154 young adults ranging from normal to pathological, and comprising 82 young adults seeking treatment for psychological distress, 26% with subthreshold symptoms and the remainder with fully syndromal psychiatric disorders, and 72 age- and gender-matched healthy young adults.

## 2 Materials and methods

### 2.1 Participants

We used the DIAMOND (Dimensions of Affect, Mood, and Neural circuitry Underlying Distress) sample (R01MH100041, PI Phillips). The available sample at the time of the analyses consisted of 170 young adults between 18 and 25 years (mean age=21.6 and standard deviation (s.d.)=2.0, 116 females). Some subjects presented signal dropout in frontal brain regions in the fMRI images, therefore in order to minimize the impact of this issue on the analysis we excluded subjects that had >15% missing voxels (voxels with NaN - Not a Number) in the contrast images that were used for the pattern regression analysis (details are described below).

A total of 154 subjects were thus included in the present study (mean age=21.6 and s.d.=2.0, 103 females). The distressed sample consisted of 82 young adults actively seeking help for psychological distress (including depressive and anxiety symptoms, and other behavioural and emotional problems such as failing to cope with everyday stressors and interpersonal relationships), irrespective of having received a DSM diagnosis. The distressed sample had a variety of current unmodified DSM-5 diagnoses, confirmed by a licensed child psychiatrist or psychologist: depressive disorder (*n*=27), anxiety disorder (*n*=43), eating disorder (*n*=3), externalizing disorder (*n*=11), trauma related disorder (*n*=9), sleep disorder (*n*=17), somato-form disorder and adjustment disorder (*n*=2). Some individuals had more than one co-morbid anxiety and/or depressive disorder. In addition, 21 distressed individuals (26%) were below threshold for any disorder. Furthermore, in the distressed sample only three participants were taking psychotropic (antidepressant) medications.

The healthy group consisted of 72 individuals (mean age=21.5 and s.d.=1.8, 47 females) not presently seeking help from such services, and with no previous personal or family history of psychiatric illness in first-degree relatives. All individuals were assessed with the Structured Clinical Interview for DSM-5, Research Version (SCID-5-RV17) before participation in the study. We ensured inclusion of a range of personality traits and behavioural problems in both groups. Participants were recruited via community advertisement, student counselling services, and a participant registry.

The exclusion criteria at screening for all participants included 1. history of head injury, neurological, pervasive developmental disorder (e.g. autism), or systemic medical disease (that could impact fMRI scans; from medical records and report by each potential participant); 2. Mini-Mental State Examination (Folstein et al., 1975) (cognitive state) score < 24; 3. premorbid NAART IQ (Blair and Spreen, 1989) estimate<85; 4. visual disturbance (<20/40 Snellen visual acuity); 5. left or mixed handedness (Annett criteria (Annett, 1970)), to ensure a uniform hemispheric dominance for interpretation of neuroimaging data; 6. Alcohol/substance use disorder (including nicotine) and/or illicit substance use (except cannabis) over the last 3 months, determined by Structured Clinical Interview for DSM5 (SCID-5) (First et al., 1995) (and psychiatric records, if available). Lifetime/present cannabis use (non substance use disorder levels) was allowed, given its common usage in 18–25 year-olds (SAMHSA, 2010). Urine tests on the scanning day excluded individuals with current illicit substance use (except cannabis, *n*=8); salivary alcohol tests excluded individuals who were intoxicated on the scanning day. Alcohol/nicotine/caffeine/cannabis use (below SCID-5-defined substance use disorder levels) per week was noted; 7. MRI exclusion criteria, including metallic foreign objects, such as aneurysm clips or pacemakers, or a questionable history of metallic fragments, proneness to panicking in enclosed spaces, and a positive pregnancy test for female individuals or self-reporting of pregnancy. The University of Pittsburgh Human Research Protection Office approved the study, and all participants provided written informed consent.

### 2.2 Symptom assessment

Anxiety and depression levels were measured using self-reported and clinician-rated measures. In order to limit the number of multiple comparisons in our analysis we used one self-reported and one clinician rated scale of anxiety and depression in the pattern regression analysis. The self-reported scales used were the *Spielberger State-Trait Anxiety Inventory* (STAI, Spielberger et al., 1983), which consists of two sessions (i.e. the trait and state sub-scales, STAI-T and STAI-S, respectively) and the depression subscale of the *Mood and Anxiety Symptom Questionnaire* (MASQ-D, Clark and Watson, 1991). The clinician-rated scales used were the *Hamilton Rating Scale for Anxiety* (HAM-A, Hamilton, 1959) and the *Hamilton Rating Scale for Depression* (HDRS*,*Hamilton, 1960; please see Greenberg et al., 2017 for all the clinical and behavioural measures collected in the DIAMOND sample). In Table 1 is presented the mean and standard deviation obtained for these scales in the considered samples. Please see full description of the scales used in this study in the Supplemental Material.

Participants' trait anxiety scores (STAI-T) ranged from 22 to 75 in the whole sample (mean=43.6 s.d.=15.0), from 25 to 75 in the distressed sample (mean=54.9 s.d.=11.0) and from 22 to 47 in the healthy sample (mean=30.6 s.d.=5.7). Participants' state anxiety scores (STAI-S) ranged from 20 to 75 in the whole sample (mean=39.0 s.d.=13.2), from 20 to 75 in the distressed sample (mean=48.0 s.d.=10.8) and from 20 to 42 in the healthy sample (mean=28.7 s.d.=6.0).

Participants' MASQ-D score ranged from 12 to 59 in the whole sample (mean=25.8 s.d.=13.4), from 17 to 59 in the distressed sample (mean=35.0 s.d.=12.0) and from 12 to 24 in the healthy sample (mean=15.3 s.d.=3.5).

Participants' HAM-A scores ranged from 0 to 27 in the whole sample (mean=6.8 s.d.=7.4; 48 zero scores), from 0 to 27 in the distressed sample (mean=12.2 s.d.=6.3; 1 zero scores) and from 0 to 6 in the healthy sample (mean=0.6 s.d=1.2; 47 zero scores).

Participants' HDRS scores ranged from 0 to 30 in the whole sample (mean=8.4 s.d.=8.6, 45 zero scores), from 2 to 30 in the distressed sample (mean=15.0 s.d.=6.0, 0 zero scores) and from 0 to 7 in the healthy sample (mean=0.8 s.d.=1.5, 45 zero scores) (Table 1).

**Table 1:** Mean and Standard Deviation of measures from whole sample, healthy sample and distressed sample.

Measures	Whole sample	Distressed sample	Healthy sample
STAI-T	43.6 (15.0)	54.9 (11.0)	30.6 (5.7)
STAI-S	39.0 (13.2)	48.0 (10.8)	28.7 (6.0)
MASQ-D	25.8 (13.4)	35.0 (12.0)	15.3 (3.5)
HDRS	8.4 (8.6)	15.0 (6.0)	0.8 (1.5)
HAM-A	6.8 (7.4)	12.2 (6.3)	0.6 (1.2)

### 2.3 fMRI paradigm

Participants completed the dynamic faces task (12 min 36 s), which has been previously described in detail (Fournier et al., 2012; Greenberg et al., 2017). Briefly, stimuli were grayscale emotional faces (happy, angry, fearful, and sad) taken from the NimStim face database (Tottenham et al., 2009), and grayscale ovals (matched in luminance with the face stimuli) that served as control stimuli. The task included three, 12-trial blocks for each emotional face type and six 6-trial blocks of shapes presented pseudo randomly. During the face trials, a face changed in emotional expression from neutral to emotional over 1 s in 5% increments. During shape trials, an oval shape changed in size to parallel the changes in the face trials. In the middle of each trial (200 to 6500 ms), a semi-transparent foreground colour flash (blue, orange or yellow) overlaid the image. Participants identified the colour of the foreground colour flash using a response pad. The neural correlates of anxiety and depression have been extensively studied using tasks that present affective stimuli such as faces with emotional expressions (Stein et al., 2007; Blair et al., 2008; Stuhrmann et al., 2011; Demenescu et al., 2011; Duval et al., 2015; Greenberg et al., 2017).

### 2.4 Image acquisition

Neuroimaging data were collected using a 3.0 Siemens Trio MRI scanner (119 subjects, distressed *n*=50 and healthy control *n*=69) and Tesla Prisma scanner (35 subjects, distressed *n* =32 and healthy control *n* =3) at the MRI Research Center at the Presbyterian Hospital, Pittsburgh. For the Siemens Trio MRI scanner, Blood‑oxygenation-level-dependent (BOLD) images were acquired with a multi-band gradient echo-planar imaging (EPI) sequence (18×(MB)3=54 slices; 2.3 mm isotropic voxels; TR/TE =1500/30 msec; Field of View=220×220 mm; matrix 96 × 96; Flip Angle 55°, Bandwidth 1860 Hz/Px). Structural 3D axial MPRAGE images were acquired in the same session (TE=3.19 ms, TR=1500 ms; Flip Angle8°; FOV=256×256 mm; 1 mm isotropic voxels; 176 continuous slices. For the Prisma scanner, structural 3D axial MPRAGE images were acquired (TR=1520 ms, TE=3.17 ms, Flip Angle 8°, FOV=256 × 256 mm, 1 mm^3^ isotropic voxels, 176 continuous slices; 4 min, 50s). BOLD functional images were acquired with a gradient echo EPI; multiband (MB)3 sequence, covering 18×(MB)3 (=54) oblique slices (TR/TE=1500/30 ms; 2.3mm^3^ slices; Flip angle=55^o^; FOV=220 × 220; Matrix=96 × 96).

### 2.5 Data pre-processing and general linear model analysis

Data were pre-processed using a combination of software packages (SPM, FSL, AFNI) implemented in Nipype (Gorgolewski et al., 2011). Pre-processing included realignment, coregistration, distortion correction, normalization, despiking, and smoothing. A first-level fixed-effect general linear model (GLM) was constructed for each participant using Statistical Parametric Mapping software, version-8 (SPM8), with the four emotion (anger, fear, sad and happy) and shape conditions. Motion parameters were included as covariates of no interest to control for participant movement. A regressor to correct for physiological fluctuations was also included, derived from the mean signal within white matter, cerebrospinal fluid and high temporal standard deviation voxels (Behzadi et al., 2007; Fournier et al., 2014). A high-pass filter (256 s), and autoregressive (AR (1)) modelling were also implemented at the first level. For each subject a contrast imaging was created to capture the overall pattern of brain activation during emotional face processing (all emotions versus shape contrast) which was used for the pattern regression analysis. Subjects were excluded if their contrast image (all emotions versus shape) contained >15% of NaN voxels (16 out of 170). Finally, a customized mask was created to include only brain voxels which were common to all participants in the contrast imaging (i.e. we excluded voxels which had a NaN in the contrast imaging for at least one participant). This criterion for creating the mask has been previously shown to improve the performance of pattern recognition analyses by decreasing the number of noninformative features/voxels in the model (Portugal et al., 2016).

### 2.6 Pattern regression analysis

Pattern regression analysis were implemented in PRoNTo (Schrouff et al., 2013a, Schrouff et al., 2013b) to investigate whether it is possible to predict anxiety and depressive scores from patterns of brain activation during dynamic emotional face processing. In the present study we used the Gaussian Process Regression, which is a probabilistic regression approach (GPR; Rasmussen and Williams, 2006). We trained and tested the GPR models based on the whole sample and based on each of the groups (for the distressed and healthy sample results please see Supplemental Material). As a comparison we also tested other pattern regression models available in PRoNTo. The results were similar across the different regression models but GPR provided slighter better results. For the sake of brevity, we included only the GPR results in the manuscript (results for the other pattern regression models can be found in the Supplementary Material).

To evaluate the GPR performance we used two different *cross-validation* strategies (a two-fold cross-validation and a five-fold cross-validation) to demonstrate that the results were not dependent on a specific cross-validation scheme. We choose two and five-fold cross validation, as these numbers of splits seemed reasonable considering our sample size. The two-fold cross-validation strategy involves dividing the data into 2 sets (*n*=77). Data from one set are left out as test samples and data from the other set are used to train the model. This procedure is then repeated, so that each data set is left out once for test. The five-fold cross validation involves dividing the data into 5 disjoint sets. Data from each set is left out once for test and data from the remaining 4 sets are used to train the model. This procedure is then repeated five times, so that each set is left out once. In both cases, the performance of the model is computed based on the concatenation of the predictions across folds, as implemented in PRoNTo v2.1. Based on the evidence from previous studies that scanner differences strongly affect images, allowing pattern classification models to predict the type of “scanner” with very high accuracy (Schrouff et al., 2013), and the evidence that it is possible to combine multi-center MRI data to create a well performing classification model if the model is trained and tested using multi-centre data (Nieuwenhuis et al., 2017), we manually balanced the proportion of data from different scanners across the different folds. Furthermore, in both cross-validation strategies we applied a *t*-test ensure that the distribution of the to-be-predicted variables (STAI-T, STAI-S, MASQ-D, HAM-A and HDRS) did not differ significantly among the folds.

The performance of the pattern regression models was measured using two metrics of agreement between the predicted and the actual scores, Pearson's correlation coefficient (r) and normalized mean squared error (MSE). The correlation coefficient (r) describes the strength of a linear relationship between two variables. A small correlation is an indication of poor predictions, and a high correlation is an indication of good prediction. The normalized MSE is the mean of the squared differences between the predicted and true scores divided by the range of predicted scores (i.e. maximum minus minimum value). It measures the error between the predicted and actual scores.

We used non-parametric permutation tests to measure the significance of the model performance. More specifically, we repeated the cross-validation procedure 1000 times and counted how many times the absolute value of the metric (r or MSE) with the permuted labels was equal to or higher than (or lower than in the case of the MSE) the one obtained with the correct labels. The p-value was then calculated by dividing this number by the number of permutations (1000). We used Bonferroni correction to account for the multiple comparisons (5 scales × 2 cross-validation strategies=10), therefore results were considered significant if the p-value<.05/10=0.005.

Age, gender and scanner were considered potential confounders that could affect the patterns of brain activity. However, removing confounds associated with the variable we want to predict (i.e. the labels) is not recommended due to the fact that this adjustment is likely to remove not only the variability in the data due to the confounds but also variability on the data associated with the labels (Rao et al., 2017; Miller and Chapman, 2001). Therefore, we used two sample *t*-tests to determine whether scanner and gender were systematically related to the clinical scores that we intended to predict and Pearson correlation to determine whether age was associated with them. We found an association between scanner and all clinical scores in the whole sample but not in the healthy and distressed cohorts when considered separately. We also found an association between gender and STAI-T, MASQ-D and HAM-A in the whole sample, distressed sample and healthy sample. There was no significant correlation between age and any of clinical variables. Due to these observed associations, we included only age as covariate/confound in the pattern regression analyses, using an approach that accounts for the training and testing separation as described in Rao and Mourao-Miranda (2017). Due to the observed association between the clinical scores and scanner and gender we cannot exclude a potential effect of these confounds on the predictive models. However, since the association between scanner and the clinical scores was not observed within the distressed sample, for the models that presented statistically significant results based on the whole sample, we repeated the analysis within the distressed sample considering age and scanner as confounds (please see results in Supplementary Material).

We computed the weight maps for the GPR models that showed statistically significant values of correlation and normalized MSE. The weight map is a spatial representation of the model's parameters or predictive function. It shows the contribution of each voxel in the image for the linear predictive function, such as the GPR with linear kernel as implemented in PRoNTo. As has been previously discussed in the literature (Schrouff et al., 2013, Schrouff et al., 2018), the weight map of linear machine learning models cannot be thresholded to make regionally specific inferences as in classical (univariate) techniques. Since each cross-validation fold yields a different weight vector, the final weight map is the average map across the folds divided by its Euclidean norm. Here, we applied a methodology, referred to as pattern localization, based on a labelled anatomical template to summarize the weight map in terms of anatomical regions (Scrouff et al., 2013, Portugal et al., 2016). Briefly, for each brain region defined by the anatomical template, the normalized weight (NW) is computed as the mean of absolute values of all voxel weights within this region divided by the number of voxels within the region. We then ranked the labelled regions according to the percentage of the total normalized weights they explained. We used the Anatomical Automatic Labeling (AAL) atlas from the WFU-PickAtlas (Maldjian et al., 2003) toolbox in SPM to define the brain regions.

### 2.7 Pattern classification analysis

In addition to the main aim of the study, which was to investigate whether wholebrain patterns of activity could be used to predict *continuous/multi-symptoms* measures associated with distress (i.e. anxiety and depression scores) in a sample ranging from normal to pathological levels of distress, we also performed a “traditional” binary classification analysis to investigate whether we could distinguish healthy vs. distressed individuals based on their the wholebrain patterns of activity. We performed a Gaussian Process Classification (GPC) in PRoNTo applying the same cross-validation schemes to the same sample used for the pattern regression analysis: 154 subjects (*n*=82 distressed youth and *n*=72 healthy individuals). The performance of the GPC was evaluated using balanced accuracy and accuracies per class. As for the GPR models, a permutation test (with 1000 permutations) was used to determine the significance of the classification performance measures.

## 3 Results

### 3.1 Pattern regression analysis

**Fig. 1:**
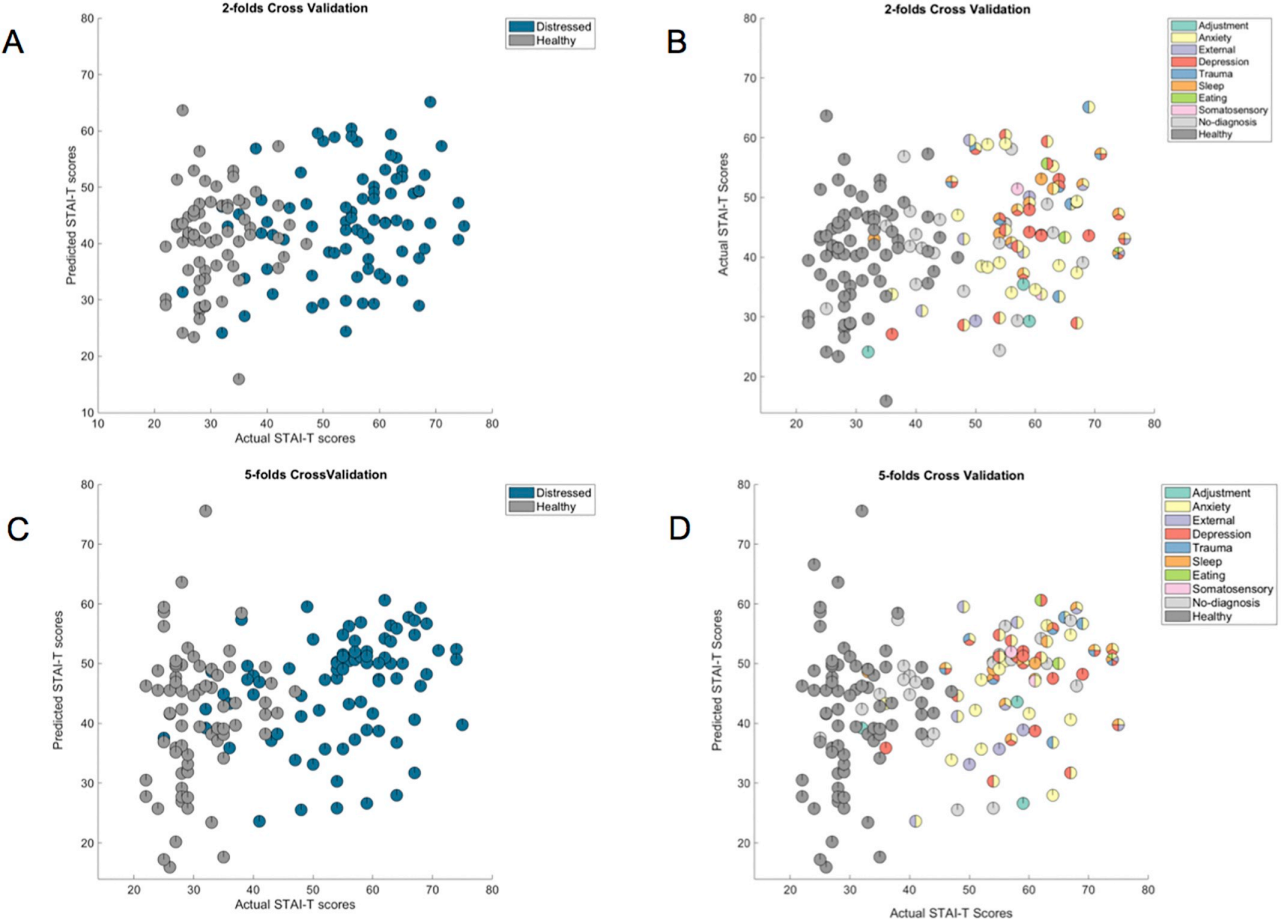
(A) Scatter plot between the actual and predicted STAI-T scores for the model based on patterns of brain activation to dynamic emotional face processing in the whole sample applying a two-folds cross-validation scheme. For visualization purposed the subjects were colour coded as belonging to the healthy and distressed samples. (B) Same plot as in (A) but for visualization purposed the subjects were colour coded according to the categorically-defined diagnoses. (C) Scatter plot between the actual and predicted STAI-T scores for the model based on patterns of brain activation to dynamic emotional face processing in the whole sample applying a five-folds cross-validation scheme. Again, for visualization purposes subjects were colour coded as belonging to the healthy and distressed samples. (D) Same plot as in (C) but for visualization purposed the subjects were colour coded according to the categorically-defined diagnoses. Distressed individuals below threshold for any disorder were labelled as ‘no-diagnosis’.

Table 2 shows the performance of the pattern regression models for predicting anxiety and depression related scores from patterns of brain activity to dynamic emotional face processing based on the whole sample. After correcting for multiple comparisons (since 5 different scales were tested using 2 cross-validation strategies the significance threshold was 0.05/10=0.005), the only significant models were the ones decoding STAI-T using both cross validation strategy (two-fold cross-validation: r=0.28 (p-value=0.001) and normalized MSE=4.47 (p-value =0.001); five-fold cross-validation: r=0.28 (p-value=0.002) and normalized MSE=4.62 (p-value=0.003); Fig. 1). These results suggest that there is an association between self-report anxiety measures and patterns of brain activity during dynamic emotional face processing in a sample that ranges from normal to pathological levels of distress, cutting across categorically-defined diagnoses. Fig. 1 shows the scatter plots between the predicted and actual STAI-T scores. For visualization purposed the subjects were colour coded as belonging to the healthy and distressed subsamples (Fig. 1A and 1C) or according to the categorically-defined diagnoses. It should be noted that 21 distressed individuals (26%) were below threshold for any psychiatric disorder (Fig. 1B and 1D). As expected, most of the distressed subjects have comorbidities. It is interesting to note that the models decoding MASQ-D almost reached significance for both cross-validations strategies, suggesting a weak association between the self-report depression measure and the patterns of brain activity during dynamic emotional face processing.

**Table 2:**
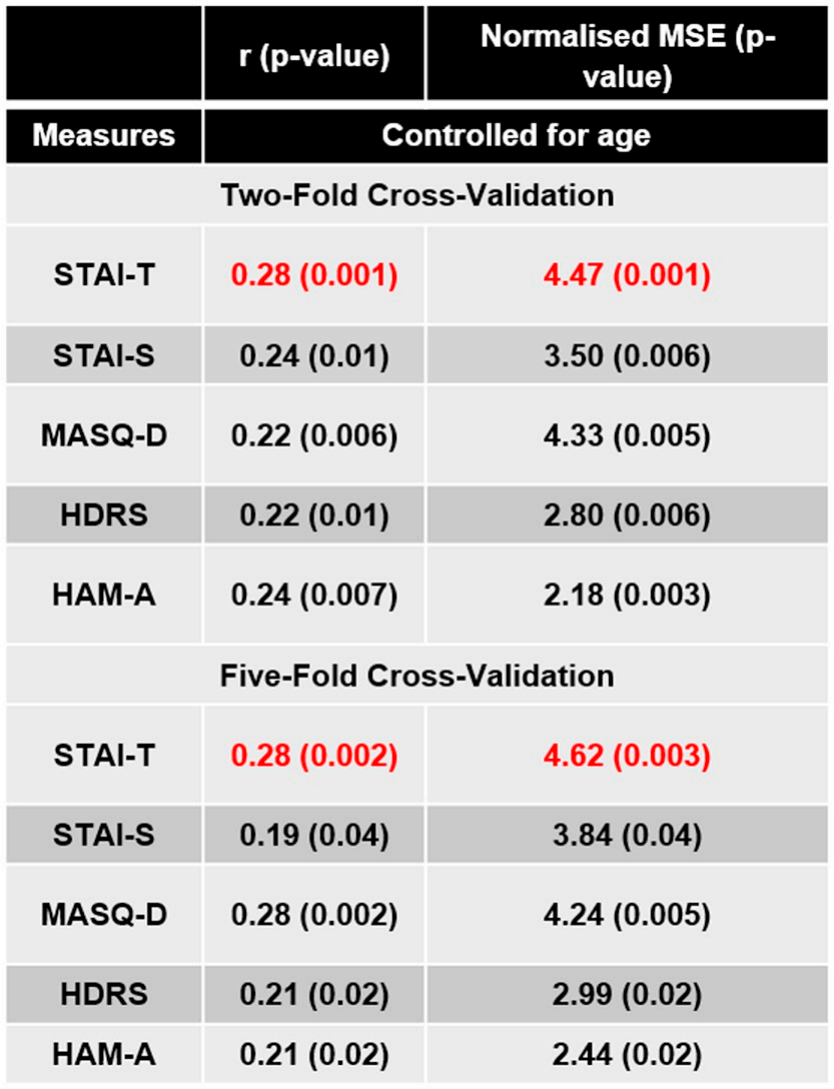
Measures of agreement between actual and decoded scores based on wholebrain activity patterns to emotional faces after controlling for covariate (age) in the whole sample. Significant results are displayed in red.

For reference: corrected p-value=0.005.

For completeness we also tested if we could predict anxiety and depression scores considering the distressed and healthy samples separately. In the distressed sample we obtained similar results as the ones obtained when considering the whole sample with almost significant p-values after correcting for multiple comparison, potentially due to the smaller sample size (Table S01 – Supplementary Material). In contrast, in the healthy sample, the models were not able to predict any of the considered clinical scores (Table S03 – Supplementary Material). These results suggest that the association between self-reported trait anxiety and brain response to dynamic emotional face processing is stronger in the distressed sample. Nevertheless, it should be noted that the variability of the STAI-T scores is much lower in the healthy sample, which potentially makes them a suboptimal target for the pattern regression model. As previously mentioned, we run an additional analysis to test whether we could still predict STAI-T scores in the distressed sample after controlling for age and scanner (within the distressed sample there was no significant association between scanner and clinical scores). The results of this model were similar to the ones obtained when controlling only by age (Table S02 – Supplementary Material).

**Fig. 2:**
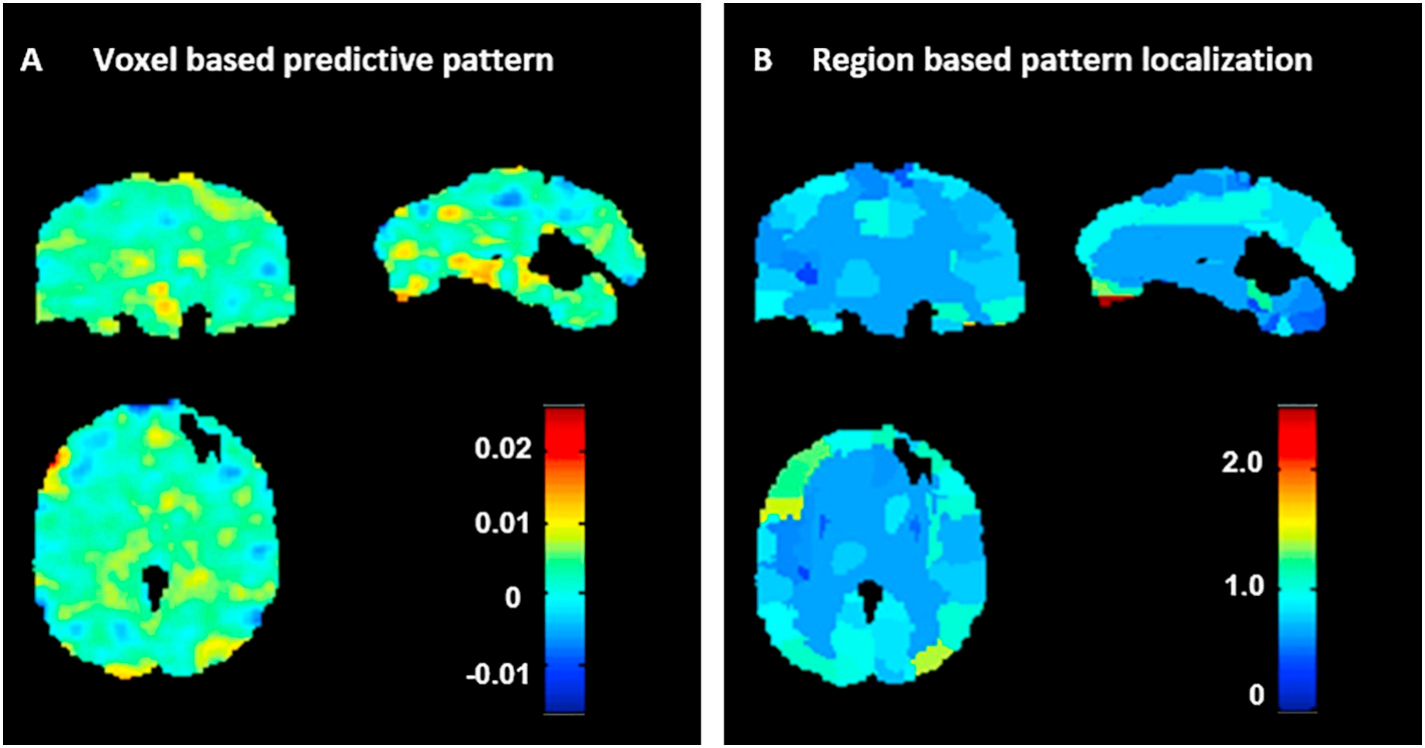
Weight maps for GPR model predicting STAI-T based on patterns of activation to dynamic emotional face processing using a two-folds cross-validation framework on the whole sample. A: Voxel-based predictive pattern. The colour bar indicates the weight of the voxels for decoding the clinical score. B: Region-based pattern localization map computed from the voxel based predictive pattern displayed in Fig. 2A. The colour bar indicates the percentage of the total normalized weights that each anatomically labelled region explains.

For the sake of brevity, we display the weight maps only for the model based on the two-fold cross-validation scheme in the main manuscript (the weight maps for the model based on the five-fold cross-validation scheme can be found in the Supplemental Material). In Fig. 2A we present the weight map for the GPR model that predicted STAI-T based on patterns of brain activation to dynamic face processing. The weight in each voxel corresponds to its contribution to the model's prediction. We emphasize that weight maps should not be interpreted as statistical parametric maps; they provide a spatial representation of the predictive function and should not be thresholded as all voxels used in the modelling contributed to the final predictions. In Fig. 2B we present the region-based pattern localization map, a post-hoc summarization map computed from the voxel based predictive pattern displayed in Fig. 2A. The colour of each region corresponds to the normalized average of voxels weights within the regions (in absolute value). The regional summarization indicate that the predictions were based on the wholebrain pattern with all regions having similar level of contribution. Table 3 shows the top 20 ranked regions according to normalized weights per region, which represent 28.6% of the total weights of the predictive function (a table showing the relative contribution of all brain regions can be found in the Supplementary Material). The regions with highest contributions were frontal and temporal regions, occipital regions and areas of the cerebellum. Note that contributions of individual regions were very small, however, suggesting that predictions were based on the overall pattern rather than on a small combination of regions. As noted in previous studies (Schrouff and Mourao-Miranda, 2018, Haufe et al., 2014, Weichwald et al., 2015, Kia et al., 2017), this however does not mean that the brain activity associated with anxiety (measured by the STAI-T score) is distributed over the wholebrain. For example, Schrouff and Mourao-Miranda (2018) have shown that when the predictive patterns are subtle (i.e. have a low signal to noise ratio) the weights are more distributed across all brain regions. Please see Table complete S04 and S05 for the complete list of regions.

**Table 3:** Top 20 ranked regions according to normalized weights per region, which represent 28.6%of the total weights of the predictive function.

Rank	Brain regions	%NW
1	Rectus_L	2.5
2	Occipital_Inf_L	1.8
3	Occipital_Inf_R	1.8
4	Rectus_R	1.7
5	Cerebelum_3_L	1.6
6	Fusiform_R	1.6
7	Cerebelum_7b_L	1.5
8	Frontal_Inf_Oper_L	1.4
9	Occipital_Mid_R	1.4
10	Temporal_Inf_R	1.3
11	Frontal_Mid_Orb_L	1.3
12	Cerebelum_4_5_L	1.3
13	Frontal_Mid_L	1.3
14	Frontal_Inf_Tri_L	1.2
15	Cerebelum_6_R	1.2
16	Vermis_3	1.2
17	Fusiform_L	1.2
18	Temporal_Pole_Mid_L	1.1
19	Frontal_Mid_Orb_R	1.1
20	Frontal_Sup_Medial_R	1.1

Abbreviations: Inf: Inferior; L: Left; Mid: Middle; Oper: Opercularis, Orb: Orbital; Post: Posterior; R: Right; Sup: Superior; Supp: Supplementary, Tri: Triangularis; % NW: Percentage of the total normalized weights that each anatomical region explains.

### 3.2 Pattern classification analysis

The GPC models were not able to accurately discriminate distressed versus healthy individuals for both cross-validation strategies (two-fold: balanced accuracy=46.19% *(*p-value=0.8); distressed accuracy=54.9% (p-value=0.6) and healthy accuracy=37.5% (p-value=0.9); five-fold: balanced accuracy=51.8% (p-value=0.35), distressed accuracy=57.7% (p-value=0.45) and healthy accuracy=45.8% (p-value=0.39)). These results are not surprising considering the high heterogeneity of the distressed sample, and show that the regression approach can be more sensitive in identifying a relationship between patterns of brain activity and continuous measures of symptom dimension within heterogeneous samples, in accord with the RDOC framework.

## 4 Discussion

The main goal of the present study was to apply pattern regression analysis to functional neuroimaging data to determine whether dimensional scales of anxiety and depression severity could be predicted from patterns of wholebrain activity in a sample of 154 young adults (aged 18–25), ranging from normal to pathological levels of distress. The GPR model was able to predict trait anxiety scores (STAI-T) from wholebrain patterns of activity to dynamic emotional face processing in a sample including distressed and healthy young adults. These results indicate an association between neural response during dynamic emotional face processing and anxiety score in the whole sample. Interestingly, when the two samples were considered separately, the GPR did not find significant results within the healthy sample but found close to significant results within the distressed sample, suggesting that the association between brain patterns of activation during dynamic face processing and self-reported anxiety is stronger in distressed individuals. The contributions of individual regions to the predictive model were very small, demonstrating that predictions were based on the overall pattern rather than on a small combination of regions. These findings represent early evidence that neuroimaging techniques may inform clinical assessment of young adults irrespective of diagnoses by allowing accurate and objective quantitative estimation of psychopathology.

In addition to the main aim of the study, we also investigated whether the patterns of wholebrain activity during dynamic face processing could be used to distinguish the distressed from the healthy individuals using Gaussian Process Classifier (GPC), with the same subjects and cross-validations strategies used for the GPR. The classification results were not significant, demonstrating that the regression approach can be more sensitive in identifying relationships between continuous dimensions of symptoms and neural measures within heterogeneous samples (ranging from normal to abnormal), supporting the RDoC framework.

Our results agree with the fact that psychiatric patients have clusters of symptoms, and that many symptoms are shared among, rather than being unique to, different psychiatric disorders. There is accumulating evidence both within and outside of the domains of depression and anxiety that supports a dimensional approach to psychopathology in which individuals' functioning is characterized along continuous measures that operationalize core psychobiological constructs (Goldberg, 2000; Widiger and Clark, 2000; Levine et al., 2001; Widiger and Samuel, 2005; Helzer et al., 2006; Insel et al., 2010; Kleinman et al., 2015; Portugal et al., 2016; MacNamara et al., 2017; Kircanski et al., 2017). For example, Macnamara et al. (2017) recently reported transdiagnostic neural correlates of anxiety and depression during affective face processing in a sample ranging from healthy individuals to individuals with psychopathology, including three primary diagnoses: social anxiety disorder (SAD), generalized anxiety disorder (GAD) or major depressive disorder (MDD) (*n* = 199). Anxiety symptom scores (HAM-A) were associated with increased activation bilaterally in the insula, in the anterior/midcingulate and in the right dorsolateral prefrontal cortex (dlPFC) in the contrast angry faces versus shapes, while depressive symptoms (HDRS) were associated with reduced right dlPFC activation in the same contrast. This study focused on finding associations between signals in individual regions and clinician rated measures using statistical univariate analysis. The present study differs from the work from Macnamara and collaborators as, instead of investigating associations between symptoms and brain activity in individual brain regions at the group level using conventional univariate analysis, we applied machine learning techniques, with the aim of determining whether measures of anxiety and depression could be predicted or decoded from patterns of wholebrain activity at the individual level. To the best of our knowledge, the present study is the first to apply pattern regression to predict individual clinical assessment in a sample of young adults including distressed and healthy individuals. Of note, our sample included a majority of female participants, which likely reflects increased help-seeking behavior in females than males.

In the present study we focus on anxiety and depression scales since it has been previously shown that the dynamic emotional face processing task elicits abnormal brain activations in individuals with anxiety and depressive disorders. Among the five scales tested (STAI-T, STAI-S, MASK-D, HDRS, HAM-S), only the sub-scale STAI-T could be significantly predicted from the wholebrain patterns of activity to dynamic emotional face processing (represented by the contrast all emotions versus shape) after correction for multiple comparisons. The *Spielberger State-Trait Anxiety Inventory* (STAI, Spielberger et al., 1983) is a self-reported scale to assess anxiety and is divided into two sub-scales: trait and state (STAI-T and STAI-S, respectively). STAI-T captures the general tendency of an individual to respond, with anxiety, to environmental stimuli. This measure shows a stable predisposition in healthy individuals and is often considered to be a risk factor for anxiety disorders and other psychiatric illnesses (Bienvenu et al., 2001; Chambers et al., 2004). Conversely, STAI-S captures a more transient response characterized by tension, apprehension, and hyperactivity of the autonomic nervous system. Our results show that there is an association between neural response to dynamic emotional face processing and trait anxiety (STAI-T) but not state anxiety (STAI-S). Furthermore, the fact that we could not predict the clinician-rated scale that measures the severity of anxiety symptoms (HAM-A), suggests that this scale is probably less associated with neural response to dynamic emotional face processing than the self-reported scale.

Interestingly, the GPR model was close to reach significant results (after correction for multiple comparison) for predicting MASQ-D, a self-reported depression scale, suggesting a weak association between neural response to dynamic emotional face processing and self-report depression. Taken together, these results suggest a potential association between emotional face processing and self-reported measures of anxiety and depression in a sample of young adults (aged 18–25), ranging from normal to pathological distress, which seems to be stronger than the association with clinician-rated measures. Nevertheless, this hypothesis needs to be confirmed in a larger sample. Our results agree with reports showing that self-reported and clinician-rated outcomes are not equivalent, with self-report and clinician ratings each providing unique information that is relevant to clinical prognosis (Uher et al., 2012). These results indicate that more neuroimaging studies should be performed in young adults (18–25-year-olds) seeking help for psychological distress irrespective of diagnosis, with the goal of identifying neurobiological measures that can predict or decode current symptomatology and potentially predict future functional outcomes which can guide appropriate choice of intervention.

Consistent with previous studies from our group the contrast between all emotion versus shape was used (Manelis et al., 2015; Hafeman et al., 2017; Greenberg et al., 2017). This contrast elicits activity in emotion processing neural regions such as bilateral amygdala, temporal and occipital fusiform cortices, frontal polar, frontal medial and orbito-frontal cortices, right ventrolateral pre-frontal cortex and right temporal polar cortex (Fusar-Poli et al., 2009; Sabatinelli et al., 2011; Manelis et al., 2015). Interestingly, the brain regions with the highest contribution to decoding the STAIT-T scores were fronto-temporal regions, occipital fusiform areas which included many neural regions elicited by this contrast in previous works. However, it is important to highlight that the contributions of individual regions to the model were very small with all brain regions having some contribution. As previously mentioned, this does not mean that the brain activity associated with anxiety is distributed over the wholebrain pattern of activity since it has been previously shown that when the predictive patterns are subtle (i.e. have a low signal to noise ratio) the weights tend to be more distributed across all brain regions (Schrouff and Mourao-Miranda, 2018).

A strength of the present study was using 18–25 year-olds seeking help for psychological distress since in our sample no one had long-term exposure to psychotropic medications. The present study was a unique opportunity to identify neuroimaging measures reflecting pathophysiologic processes in individuals seeking help for psychological distress, without the potential confounds of “scarring” effects resulting from factors associated with having a long history of seeking help for mental health problems in adulthood. Furthermore, studying individuals between 18 and 25 years is a unique opportunity to identify dimensions of pathophysiology at a critical developmental period when brain development is still occurring, so that appropriate, biologically-informed interventions have potential to take advantage of the plasticity of the brain during this developmental period to minimize, or even prevent, long-term abnormalities in neural circuitry, and chronic, recurrent, or difficult to treat mental health problems.

There were also some limitations in the present study. The main limitation was the fact that two potential confounders (scanner and gender) were associated with the clinical scores we wanted to predict. Therefore, removing their effect from patterns of brain activity would also remove the variability in the data associated with the clinical scores. In order to address this limitation, we performed additional analyses to investigate the effect of controlling by scanner and age on the distressed sample (which had no association between scanners and clinical scores). We obtained similar results as the ones obtained on the distressed sample after controlling only by age. The manual matching of subjects across the different cross-validation splits (to keep balanced proportions of scanner and gender) might also affect the results. Therefore, we used two cross-validation schemes to show that the results were not due to a specific data split. Another limitation of the study was the fact that the GPR predictive patterns were difficult to interpret in terms of the underlying neurobiology. The models' weights where distributed across the wholebrain and there was no evidence of specific brain regions having stronger contributions to the predictions. In addition, given the nature of contrast data used (all faces versus shape), we cannot exclude the fact that our results could be driven by face processing in general (not by emotional faces in particular) or by a process occurring during shape processing. Finally, even though we used two cross validation schemes (two-fold (or half split) cross-validation and five-fold cross-validation), ideally, predictive models should be further validated with a truly independent sample.

## 5 Future directions and conclusion

The present study was a proof of concept study designed to examine whether pattern regression analysis could be applied to neuroimaging data to predict individual-level severity along dimensions of anxiety and depression in young adults with symptomatology that varies from normal to pathological, irrespective of primary diagnoses in a research domain framework. The fact that the GPR model was able to decode the STAI-T score from patterns of brain activation in our heterogeneous sample (including healthy and distressed individuals) is evidence that there was a common underlying pattern of brain activation during dynamic face processing in the sample that was associated with a continuous measure of anxiety trait (operationalized as STAI-T scores). Our results support the research domain criteria (RDoC) recommendation, which advocates that it is critical to study heterogeneous samples to identify biomarkers reflecting pathophysiological processes, irrespective of diagnosis.

Moving forward, similar approaches could be applied to investigate whether other clinical scales can be predicted from wholebrain patterns of activity during other emotional and/or cognitive processes (e.g. reward and n-back tasks). Taken together, we advocate adopting a multi-dimensional assessment strategy for investigating the nature of pathophysiology of mental disorders and improving therapeutics since neuroimaging measures can ultimately provide biological targets for novel treatment development for this vulnerable population. Future studies, using a combination of multimodal neuroimaging and non-imaging information, can build on the present findings to determine the extent to which individual-level patterns of neural function either instead of or in combination with clinical scores, familial and demographic measures can predict individual-level future clinical outcomes on sufficiently large datasets. Furthermore, with the technological advances enabling acquisition of large databases of patients and healthy subjects, machine learning represents a powerful tool in the search for psychiatric biomarkers.

## Appendix A Supplementary data

Supplementary material Image 1
