# Supplementary material for: Predicting anxiety from wholebrain activity patterns to emotional faces in young adults: a machine learning approach
Source: Neuroimage Clin. 2019 Apr 3;23:101813. doi: 10.1016/j.nicl.2019.101813 (PMC6517640; doi:10.1016/j.nicl.2019.101813)
Supplement: Supplementary file 1 — Supplementary material [file mmc1.docx]

**Supplementary Material**

**Psychometrics**

The *Spielberger State-Trait Anxiety Inventory* (STAI, Spielberger et al., 1983) scale consists of a four-point Likert scale to assess anxiety. This scale is divided into two sections: trait (STAI-T) and state scale (STAI-S), each with 20 questions. Trait anxiety is a self-report questionnaire that consists of 20 items ranging from 20 to 80 that represents a general tendency an individual has to respond with anxiety in response to environmental stimuli. State anxiety is a transient condition characterized by tension, apprehension, and hyperactivity of the autonomic nervous system.

The *General Distress: Depressive Symptoms scale* (MASQ-D) consists of 12 items and contains several items reflecting depressed mood along with other relatively nonspecific symptoms of mood disorder (e.g., feelings of disappointment and failure, self-blame, pessimism, Clark et al., 1991).

The *Hamilton Rating Scale for anxiety* ((HAM-A, Hamilton, 1959) is a clinician-rated scale that measures the severity of anxiety symptoms. The HAM-A consists of 14 items, each defined by a series of symptoms, and measures both psychic anxiety (i.e., mental agitation and psychological distress) and somatic anxiety (i.e., physical complaints related to anxiety).

The *Hamilton Rating Scale for depression* (HDRS, Hamilton, 1960) is a clinical rated scale that measures the severity of depressive symptoms. Although the HDRS form consists of 21 items, the scoring is based on the first 17 items.

**Pattern Regression Analysis Based on the Distressed Sample**

In order to investigate whether a minimum of symptoms observed in the distressed sample could improve or explain the performance of the pattern regression models, we tested whether we could predict anxiety and depression scores considering only the distressed sample. The best model was the one predicting STAI-T (r=0.32, p-value = 0.0004 and MSE =125, p-value = 0.009, Table S-01) from the patterns of brain activation, however the results were not significant after correction for multiple comparisons, potentially due to the smaller sample size.

Furthermore, in order to investigate the potential effect of scanner on the predictive models we run an additional model for predicting STAI-T within the distressed sample controlling by scanner and age. In contrast with the whole sample, there was no association between scanners and STAI-T within the distressed sample. We obtained similar results as the ones obtained on the distressed sample after controlling only by age (Table S04).

Note that since 5 different scales were tested using 2 cross-validation strategies controlling per scanner and in addition 2 more tests were performed controlling for age and scanner, the significance threshold was 0.05/12=0.004.

**Table S01:** Measures of agreement between actual and decoded scores based on functional neuroimaging scans (all faces versus shape contrast) after controlling for covariate (age) in the distressed sample. No results were significant after correcting for multiple comparisons.

|  | **r (p value)** | **Norm MSE (p value)** |
| --- | --- | --- |
| **Measures** | **Controlled for age** | |
| **Two-Fold Cross Validation** | | |
| **STAI-T** | **0.32 (0.004)** | **2.49 (0.005)** |
| **STAI-S** | **0.14 (0.14)** | **2.84 (0.26)** |
| **MASQ-D** | **0.29 (0.007)** | **3.47 (0.02)** |
| **HDRS** | **0.12 (0.17)** | **2.06 (0.45)** |
| **HAM-A** | **0.05 (0.33)** | **2.05 (0.35)** |
| **Five-Fold Cross Validation** | | |
| **STAI-T** | **0.30 (0.009)** | **123 (0.005)** |
| **STAI-S** | **0.11 (0.16)** | **3.04 (0.34)** |
| **MASQ-D** | **0.16 (0.08)** | **4.05 (0.13)** |
| **HDRS** | **0.03 (0.39)** | **2.37 (0.70)** |
| **HAM-A** | **-0.08 (0.69)** | **2.36 (0.64)** |

**For reference: corrected p-value=0.004**

**Table S02:** Measures of agreement between actual and decoded scores based on functional neuroimaging scans (all faces versus shape contrast) after controlling for covariate (age and scanner) in the distressed sample.

|  | **r (p value)** | **Norm MSE (p value)** |
| --- | --- | --- |
| **Measures** | **Controlled for age and scanner** | |
| **Two-Fold Cross Validation** | | |
| **STAI-T** | **0.33 (0.004)** | **2.4 (0.003)** |
| **Five-Fold Cross Validation** | | |
| **STAI-T** | **0.27 (0.02)** | **2.5 (0.02)** |

For reference: corrected p-value=0.004

**Pattern Regression Analysis Based on the Healthy Sample**

For completeness we also tested if we could predict anxiety and depression scores considering only the healthy sample. In the healthy sample, the models were not able to predict any of the considered clinical scores (Table S-03). These results suggest that within the healthy subjects the association between the brain response to dynamic emotional face processing and anxiety and depression scores might be absent. Please note that the variability of the considered scores is much lower for the healthy sample. In this case the scores might not be good target for the pattern regression model.

**Table S03:** Measures of agreement between actual and decoded scores based on functional neuroimaging scans (all faces versus shape contrast) after controlling for covariate (age) in the healthy sample.

|  | **r (p value)** | **Normd MSE (p value)** |
| --- | --- | --- |
| **Measures** | **Controlled for age** | |
| **Two-Fold Cross Validation** | | |
| **STAI-T** | **-0.01 (0.51)** | **1.7 (0.41)** |
| **STAI-S** | **0.05 (0.35)** | **1.9 (0.14)** |
| **MASQ-D** | **-0.06 (0.63)** | **1.0 (0.64)** |
| **HDRS** | **-0.14 (0.72)** | **0.5 (0.81)** |
| **HAM-A** | **-0.18 (0.85)** | **0.4 (0.80)** |
| **Five-Fold Cross Validation** | | |
| **STAI-T** | **0.05 (0.34)** | **1.9 (0.58)** |
| **STAI-S** | **0.06 (0.32)** | **2.0 (0.25)** |
| **MASQ-D** | **-0.04 (0.60)** | **1.2 (0.82)** |
| **HDRS** | **0.03 (0.35)** | **0.4 (0.33)** |
| **HAM-A** | **-0.24 (0.94)** | **0.4 (0.86)** |

For reference: corrected p-value=0.005

**Voxel-Based Predictive Patterns**

In FigS1A we present the weight map for the GPR model that predicted STAI-T based on patterns of brain activation to dynamic emotional face processing using the five-folds cross-validation procedure on the whole sample. Fig S1B depicts the region-based pattern localization map (computed from the voxel based predictive pattern displayed in Fig S1A). The colour of each region corresponds to the normalized average of voxels weights within the regions (in absolute value). Similarly, to the results observed using the two-folds cross-validation scheme, the regional summarization indicates that the predictive pattern is very distributed across the brain, i.e. all regions contributed with similar weights for the decision function.

**
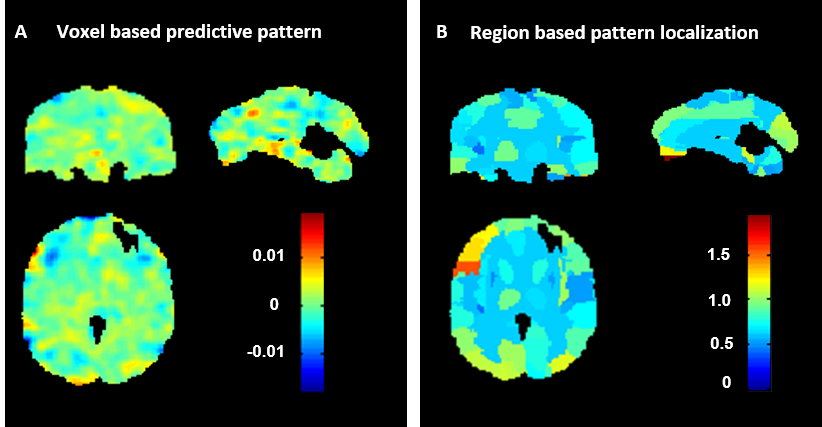
**

**Figure S01: Weight maps for GPR model predicting STAI-T based on patterns of activation to dynamic emotional face processing using a five-fold cross-validation framework on the whole sample**. **A:** Voxel-based predictive pattern. The colour bar indicates the weight of the voxels for decoding the clinical score. The colour bar indicates the voxels weight. **B:** Region-based pattern localization map computed from the voxel based predictive pattern displayed in Fig S01A. The colour bar indicates the percentage of the total normalized weights that each anatomically labelled region explains.

Table S04 shows the complete list of regions ranked according to normalized weights per region for the model predicting STAT-T based on the whole sample using the five-folds cross validation scheme. As we can see the top 20 ranked regions according to normalized weights per region, which represent 28.5% of the total weights of the prediction function, were very similar to the model using the two-folds cross-validation scheme which includes frontal e temporal regions, occipital regions and areas of the cerebellum.

**Table S04** complete list of regions ranked according to normalized weights per region for the model predicting STAT-T from patterns of wholebrain activation to dynamic emotional face processing based on the whole sample using the 5-folds cross validation scheme.

| **Rank** | **Brain Regions** | **%NW** |
| --- | --- | --- |
| **1** | **Rectus_L** | **2.0** |
| **2** | **Temporal_Pole_Mid_L** | **2.0** |
| **3** | **Rectus_R** | **1.7** |
| **4** | **Occipital_Inf_L** | **1.7** |
| **5** | **Occipital_Inf_R** | **1.6** |
| **6** | **Cerebelum_7b_L** | **1.6** |
| **7** | **Frontal_Inf_Oper_** | **1.5** |
| **8** | **Cerebelum_3_L** | **1.5** |
| **9** | **Temporal_Pole_Sup_L** | **1.4** |
| **10** | **Fusiform_R** | **1.4** |
| **11** | **Cerebelum_Crus1_L** | **1.3** |
| **12** | **Temporal_Inf_R** | **1.3** |
| **13** | **Frontal_Inf_Tri_L** | **1.3** |
| **14** | **Frontal_Mid_Orb_R** | **1.2** |
| **15** | **Fusiform_L** | **1.2** |
| **16** | **Frontal_Mid_L** | **1.2** |
| **17** | **Frontal_Mid_Orb_L** | **1.2** |
| **18** | **Cerebelum_6_R** | **1.2** |
| **19** | **Occipital_Mid_R** | **1.1** |
| **20** | **Vermis_3** | **1.1** |
| **21** | **Cerebelum_Crus1_R** | **1.1** |
| **22** | **Occipital_Sup_L** | **1.1** |
| **23** | **Cerebelum_4_5_L** | **1.1** |
| **24** | **Occipital_Mid_L** | **1.1** |
| **25** | **Cuneus_L** | **1.1** |
| **26** | **Parietal_Sup_R** | **1.0** |
| **27** | **Lingual_R** | **1.0** |
| **28** | **Temporal_Inf_L** | **1.0** |
| **29** | **Insula_R** | **1.0** |
| **30** | **Calcarine_L** | **1.0** |
| **31** | **Angular_L** | **1.0** |
| **32** | **Hippocampus_R** | **1.0** |
| **33** | **Frontal_Mid_R** | **1.0** |
| **34** | **Temporal_Mid_L** | **1.0** |
| **35** | **Amygdala_L** | **1.0** |
| **36** | **Angular_R** | **1.0** |
| **37** | **Temporal_Pole_Mid_R** | **1.0** |
| **38** | **Vermis_9** | **0.9** |
| **39** | **Frontal_Sup_Medial_L** | **0.9** |
| **40** | **Lingual_L** | **0.9** |
| **41** | **Thalamus_L** | **0.9** |
| **42** | **Frontal_Sup_R** | **0.9** |
| **43** | **Cingulum_Mid_R** | **0.9** |
| **44** | **Frontal_Sup_Medial_R** | **0.9** |
| **45** | **Frontal_Sup_L** | **0.9** |
| **46** | **Frontal_Inf_Oper_R** | **0.9** |
| **47** | **Cerebelum_Crus2_L** | **0.9** |
| **48** | **Cerebelum_3_R** | **0.9** |
| **49** | **Cingulum_Mid_L** | **0.9** |
| **50** | **Precentral_L** | **0.9** |
| **51** | **Frontal_Mid_Orb_L** | **0.9** |
| **52** | **Frontal_Inf_Tri_R** | **0.9** |
| **53** | **Temporal_Mid_R** | **0.9** |
| **54** | **Cerebelum_4_5_R** | **0.9** |
| **55** | **Parietal_Sup_L** | **0.9** |
| **56** | **Precentral_R** | **0.9** |
| **57** | **Frontal_Sup_Orb_R** | **0.8** |
| **58** | **Cerebelum_9_L** | **0.8** |
| **59** | **Occipital_Sup_R** | **0.8** |
| **60** | **Precuneus_R** | **0.8** |
| **61** | **Temporal_Pole_Sup_R** | **0.8** |
| **62** | **Frontal_Inf_Orb_R** | **0.8** |
| **63** | **Parietal_Inf_R** | **0.8** |
| **64** | **Temporal_Sup_L** | **0.8** |
| **65** | **Calcarine_R** | **0.8** |
| **66** | **Postcentral_L** | **0.8** |
| **67** | **Cerebelum_6_L** | **0.8** |
| **68** | **Vermis_6** | **0.8** |
| **69** | **Supp_Motor_Area_R** | **0.8** |
| **70** | **Caudate_R** | **0.8** |
| **71** | **Amygdala_R** | **0.7** |
| **72** | **Cerebelum_8_L** | **0.7** |
| **73** | **Frontal_Inf_Orb_L** | **0.7** |
| **74** | **Caudate_L** | **0.7** |
| **75** | **Cingulum_Post_R** | **0.7** |
| **76** | **SupraMarginal_R** | **0.7** |
| **77** | **Postcentral_R** | **0.7** |
| **78** | **Cuneus_R** | **0.7** |
| **79** | **Temporal_Sup_R** | **0.7** |
| **80** | **ParaHippocampal_L** | **0.7** |
| **81** | **Insula_L** | **0.7** |
| **82** | **Parietal_Inf_L** | **0.7** |
| **83** | **Cingulum_Ant_L** | **0.7** |
| **84** | **Hippocampus_L** | **0.7** |
| **85** | **Thalamus_R** | **0.6** |
| **86** | **Cingulum_Post_L** | **0.6** |
| **87** | **Cerebelum_9_R** | **0.6** |
| **88** | **SupraMarginal_L** | **0.6** |
| **89** | **Frontal_Mid_Orb_R** | **0.6** |
| **90** | **Precuneus_L** | **0.6** |
| **91** | **Vermis_4_5** | **0.6** |
| **92** | **Pallidum_L** | **0.6** |
| **93** | **Others** | **0.6** |
| **94** | **Cingulum_Ant_R** | **0.6** |
| **95** | **Rolandic_Oper_L** | **0.6** |
| **96** | **Supp_Motor_Area_L** | **0.6** |
| **97** | **Olfactory_R** | **0.6** |
| **98** | **Vermis_7** | **0.6** |
| **99** | **Paracentral_Lobule_L** | **0.6** |
| **100** | **Heschl_R** | **0.5** |
| **101** | **Vermis_1_2** | **0.5** |
| **102** | **Vermis_10** | **0.5** |
| **103** | **Frontal_Sup_Orb_L** | **0.5** |
| **104** | **Rolandic_Oper_R** | **0.5** |
| **105** | **Putamen_R** | **0.5** |
| **106** | **ParaHippocampal_R** | **0.5** |
| **107** | **Vermis_8** | **0.5** |
| **108** | **Olfactory_L** | **0.5** |
| **109** | **Cerebelum_Crus2_R** | **0.5** |
| **110** | **Paracentral_Lobule_R** | **0.5** |
| **111** | **Putamen_L** | **0.5** |
| **112** | **Cerebelum_8_R** | **0.4** |
| **113** | **Heschl_L** | **0.4** |
| **114** | **Pallidum_R** | **0.4** |

Abbreviations: Inf: Inferior; L: Left; Mid: Middle; Oper: Opercularis, Orb: Orbital; Post: Posterior; R: Right; Sup: Superir; Supp: Supplementary, Tri: Triangularis; % NW: Percentage of the total normalized weights that each anatomical region explains.

**Table S05** complete list of regions ranked according to normalized weights per region for the model predicting STAI-T from patterns of whole brain activation to dynamic emotional face processing based on the whole sample using the 2-folds cross validation scheme.

| **Rank** | **Brain Regions** | **%NW** |
| --- | --- | --- |
| **1** | **Rectus_L** | **2.5** |
| **2** | **Occipital_Inf_L** | **1.8** |
| **3** | **Occipital_Inf_R** | **1.8** |
| **4** | **Rectus_R** | **1.8** |
| **5** | **Cerebelum_3_L** | **1.7** |
| **6** | **Fusiform_R** | **1.6** |
| **7** | **Cerebelum_7b_L** | **1.5** |
| **8** | **Frontal_Inf_Oper_L** | **1.4** |
| **9** | **Occipital_Mid_R** | **1.4** |
| **10** | **Temporal_Inf_R** | **1.4** |
| **11** | **Frontal_Mid_Orb_L** | **1.3** |
| **12** | **Cerebelum_4_5_L** | **1.3** |
| **13** | **Frontal_Mid_L** | **1.3** |
| **14** | **Frontal_Inf_Tri_L** | **1.2** |
| **15** | **Cerebelum_6_R** | **1.2** |
| **16** | **Vermis_3** | **1.2** |
| **17** | **Fusiform_L** | **1.2** |
| **18** | **Temporal_Pole_Mid_L** | **1.1** |
| **19** | **Frontal_Mid_Orb_R** | **1.1** |
| **20** | **Frontal_Sup_Medial_R** | **1.1** |
| **21** | **Frontal_Mid_R** | **1.1** |
| **22** | **Cerebelum_Crus1_L** | **1.1** |
| **23** | **Hippocampus_R** | **1.1** |
| **24** | **Occipital_Mid_L** | **1.1** |
| **25** | **Parietal_Sup_R** | **1.1** |
| **26** | **Amygdala_L** | **1.0** |
| **27** | **Insula_R** | **1.0** |
| **28** | **Temporal_Mid_R** | **1.0** |
| **29** | **Parietal_Sup_L** | **1.0** |
| **30** | **Frontal_Inf_Oper_R** | **1.0** |
| **31** | **Cerebelum_4_5_R** | **1.0** |
| **32** | **Angular_R** | **1.0** |
| **33** | **Occipital_Sup_L** | **1.0** |
| **34** | **Parietal_Inf_R** | **1.0** |
| **35** | **Cuneus_L** | **1.0** |
| **36** | **Cingulum_Mid_L** | **1.0** |
| **37** | **Frontal_Sup_L** | **1.0** |
| **38** | **Frontal_Inf_Tri_R** | **1.0** |
| **39** | **Temporal_Pole_Sup_L** | **1.0** |
| **40** | **Cerebelum_3_R** | **0.9** |
| **41** | **Frontal_Inf_Orb_R** | **0.9** |
| **42** | **Frontal_Sup_Medial_L** | **0.9** |
| **43** | **Calcarine_L** | **0.9** |
| **44** | **Cerebelum_Crus1_R** | **0.9** |
| **45** | **Lingual_R** | **0.9** |
| **46** | **Precentral_L** | **0.9** |
| **47** | **Lingual_L** | **0.9** |
| **48** | **Frontal_Mid_Orb_L** | **0.9** |
| **49** | **Angular_L** | **0.9** |
| **50** | **Cingulum_Post_L** | **0.9** |
| **51** | **Temporal_Mid_L** | **0.9** |
| **52** | **Occipital_Sup_R** | **0.9** |
| **53** | **Precuneus_R** | **0.9** |
| **54** | **ParaHippocampal_L** | **0.9** |
| **55** | **Temporal_Pole_Mid_R** | **0.9** |
| **56** | **Precentral_R** | **0.9** |
| **57** | **Caudate_R** | **0.8** |
| **58** | **Vermis_9** | **0.8** |
| **59** | **Postcentral_L** | **0.8** |
| **60** | **Cingulum_Post_R** | **0.8** |
| **61** | **Calcarine_R** | **0.8** |
| **62** | **Frontal_Sup_Orb_R** | **0.8** |
| **63** | **Precuneus_L** | **0.8** |
| **64** | **Cerebelum_6_L** | **0.8** |
| **65** | **Cingulum_Mid_R** | **0.8** |
| **66** | **Temporal_Pole_Sup_R** | **0.8** |
| **67** | **Heschl_R** | **0.8** |
| **68** | **ParaHippocampal_R** | **0.8** |
| **69** | **Thalamus_L** | **0.8** |
| **70** | **SupraMarginal_R** | **0.8** |
| **71** | **Frontal_Sup_R** | **0.8** |
| **72** | **Parietal_Inf_L** | **0.7** |
| **73** | **Temporal_Sup_R** | **0.7** |
| **74** | **Temporal_Sup_L** | **0.7** |
| **75** | **Cuneus_R** | **0.7** |
| **76** | **Cerebelum_8_L** | **0.7** |
| **77** | **Frontal_Inf_Orb_L** | **0.7** |
| **78** | **Hippocampus_L** | **0.7** |
| **79** | **Amygdala_R** | **0.7** |
| **80** | **Supp_Motor_Area_R** | **0.7** |
| **81** | **Cerebelum_9_L** | **0.7** |
| **82** | **Frontal_Mid_Orb_R** | **0.7** |
| **83** | **Pallidum_L** | **0.7** |
| **84** | **Rolandic_Oper_R** | **0.7** |
| **85** | **Postcentral_R** | **0.7** |
| **86** | **Vermis_4_5** | **0.6** |
| **87** | **Cingulum_Ant_L** | **0.6** |
| **88** | **Caudate_L** | **0.6** |
| **89** | **Cerebelum_Crus2_L** | **0.6** |
| **90** | **Others** | **0.6** |
| **91** | **Thalamus_R** | **0.6** |
| **92** | **Supp_Motor_Area_L** | **0.6** |
| **93** | **Temporal_Inf_L** | **0.6** |
| **94** | **SupraMarginal_L** | **0.6** |
| **95** | **Cingulum_Ant_R** | **0.6** |
| **96** | **Insula_L** | **0.6** |
| **97** | **Cerebelum_9_R** | **0.6** |
| **98** | **Olfactory_L** | **0.6** |
| **99** | **Paracentral_Lobule_L** | **0.6** |
| **100** | **Rolandic_Oper_L** | **0.5** |
| **101** | **Vermis_6** | **0.5** |
| **102** | **Vermis_8** | **0.5** |
| **103** | **Putamen_R** | **0.5** |
| **104** | **Frontal_Sup_Orb_L** | **0.5** |
| **105** | **Pallidum_R** | **0.5** |
| **106** | **Putamen_L** | **0.5** |
| **107** | **Vermis_10** | **0.4** |
| **108** | **Paracentral_Lobule_R** | **0.4** |
| **109** | **Vermis_7** | **0.4** |
| **110** | **Cerebelum_Crus2_R** | **0.4** |
| **111** | **Others** | **0.4** |
| **112** | **Cerebelum_8_R** | **0.4** |
| **113** | **Vermis_1_2** | **0.4** |
| **114** | **Heschl_L** | **0.4** |

Abbreviations: Inf: Inferior; L: Left; Mid: Middle; Oper: Opercularis, Orb: Orbital; Post: Posterior; R: Right; Sup: Superir; Supp: Supplementary, Tri: Triangularis; % NW: Percentage of the total normalized weights that each anatomical region explains.

**Comparison with other pattern regression approaches**

**Table S06:** Measures of agreement between actual and decoded STAI-T scores based on functional neuroimaging scans (all faces versus shape contrast) after controlling for covariate (age) in the whole sample.

|  | **r (p value)** | **Norm MSE (p value)** |
| --- | --- | --- |
| **Measures** | **Controlled for age and scanner** | |
| **Two-Fold Cross Validation** | | |
| **KRR** | **0.28 (0.002)** | **4.5 (0.002)** |
| **MKL** | **0.22 (0.01)** | **4.9 (0.003)** |

**References**

1. Clark LA and Watson D. (1991) Tripartite model of anxiety and depression: psychometric evidence and taxonomic implications. J Abnorm Psychol; 100(3):316-36. PMID:1918611 [DOI: 10.1037/0021-843X.100.3.316](http://psycnet.apa.org/doi/10.1037/0021-843X.100.3.316)
2. Hamilton M. (1959) The assessment of anxiety states by rating. Br J Med Psychol; 32:50–55. PMID:13638508 [DOI: 10.1111/j.2044-8341.1959.tb00467.x](http://psycnet.apa.org/doi/10.1111/j.2044-8341.1959.tb00467.x)
3. Hamilton M. (1960) A rating scale for depression. J Neurol Neurosurg Psychiatry; 23:56–62. PMID:14399272 [DOI:10.1136/jnnp.23.1.56](http://dx.doi.org/10.1136/jnnp.23.1.56)
4. Spielberger CD, Gorsuch RL, Lushene R. (1983) State-Trait Anxiety Invenstory Test Manual Form Y. Consulting Psychological Press: Palo Alto, CA.
